# Dietary sodium hyaluronate and eggshell membrane improve skin barrier function and coat health in Ragdoll cats

**DOI:** 10.3389/fvets.2026.1882271

**Published:** 2026-07-27

**Authors:** Qianqian Chen, Weiwei Wang, Huakai Wang, Mengyao Ma, Chengyi Miao, Yuqiang Zhang, Shanyu Lei, Li Pan, Yiran Liu, Xiaohan Chang, Jianmei Wang, Wei Xiong

**Affiliations:** 1 Food Laboratory of Zhongyuan, Luohe, China; 2 College of Animal Science and Technology, China Agricultural University, Beijing, China

**Keywords:** Ragdoll cat, sodium hyaluronate, eggshell membrane, skin barrier, coat quality, antioxidant status, fecal microbiota

## Abstract

**Introduction:**

Skin and coat health are important indicators of feline well-being and are influenced by epidermal barrier integrity, nutritional status, oxidative balance, and microbial homeostasis. However, the effects of dietary sodium hyaluronate (SH) and eggshell membrane (ESM), either alone or in combination, remain insufficiently characterized in cats.

**Methods:**

Twenty-four healthy 6-month-old Ragdoll cats were randomly assigned to four dietary groups (*n* = 6 per group): a basal-diet control, 250 mg/kg SH, 250 mg/kg ESM, or 125 mg/kg SH plus 125 mg/kg ESM. After 56 days of supplementation, hair microstructure, coat sensory and colorimetric traits, transepidermal water loss (TEWL), hematological and serum biomarkers, hair nutrient composition, and fecal microbiota were evaluated.

**Results:**

Dietary treatment did not significantly affect body weight. The combined SH + ESM treatment was associated with the most consistent improvements in hair cuticle morphology, hair fiber diameter, coat gloss, compliance, skin elasticity, coat brightness, and TEWL. It was also associated with lower serum malondialdehyde and interleukin-31 concentrations, higher selected antioxidant indices, increased serum zinc, copper, and vitamin E concentrations, and increased deposition of zinc and several amino acids in hair. Alpha diversity of the fecal microbiota was not significantly altered, whereas taxonomic composition differed among treatments, with greater relative abundances of Blautia and Lachnospiraceae-associated taxa in the combined-treatment group.

**Discussion:**

Combined SH and ESM supplementation was associated with broader skin- and coat-related responses than either ingredient alone. These findings support its potential as a nutritional strategy for maintaining feline skin and coat health; however, the relatively small cohort of healthy juvenile cats and the exploratory nature of the microbiota analyses limit causal and clinical inference.

## 1 Introduction

Skin and coat health are among the most visible indicators of well-being in companion cats, but they are also biologically complex traits influenced by epidermal barrier integrity, immune regulation, and microbial homeostasis. In feline dermatology, allergic and inflammatory skin disorders remain common, and current evidence suggests that feline skin disease should be understood not simply as a superficial cosmetic problem, but as a multifactorial condition involving barrier dysfunction, immune dysregulation, and microbiome disturbance (1–3). Even in clinically healthy cats, suboptimal coat gloss, scaling, roughness, or increased transepidermal water loss (TEWL) may reflect early alterations in skin homeostasis. Accordingly, nutritional intervention has garnered growing interest as a practical strategy for maintaining skin and coat quality, as well as supporting long-term dermatological health in cats (4). A meaningful evaluation of feline skin and coat health should go beyond appearance alone. TEWL is widely used as an objective indicator of skin barrier function, and feline studies have shown that TEWL can reflect barrier disruption and correlate with lesion severity in cats (5, 6). At the same time, coat quality is also shaped by nutrient availability for hair-fiber formation and by trace mineral status. Recent feeding work in adult cats further showed that the source of supplemental trace minerals can influence hair loss, dander, and overall haircoat characteristics, supporting the view that nutritional modulation can affect measurable coat outcomes rather than appearance alone (7). These findings indicate that studies of feline skin and coat nutrition should ideally combine barrier-related, structural, and nutritional endpoints within an integrated evaluation framework.

Sodium Hyaluronate (SH) and eggshell membrane (ESM) are two promising ingredients with potentially complementary roles in skin support. SH is a key extracellular-matrix component involved in hydration and tissue viscoelasticity, and recent clinical studies have shown that oral SH-based supplementation can improve skin brightness, hydration, smoothness, and related appearance parameters (8, 9). ESM, in contrast, is rich in collagen-associated and glycosaminoglycan-related components and has recently been reported to improve skin hydration, elasticity, TEWL-related parameters, and biomechanical properties in randomized clinical trials (10, 11). Collectively, available evidence suggests that SH may be more closely related to hydration and matrix support, whereas ESM may provide stronger structural and biomechanical support. However, evidence for either ingredient in cats remains limited, and no study has yet compared the effects of supplementation with SH alone, ESM alone, or a combination of both on feline skin and coat health.

Another important concept relevant to skin-directed nutrition is the gut–skin axis. In dogs and cats, diet is a major determinant of gut microbial composition, and growing evidence indicates that microbiome-targeted nutritional strategies may influence host homeostasis and disease management (12, 13). This is particularly relevant in dermatology because microbial alterations have increasingly been discussed in relation to skin health and disease in cats (3). Therefore, in addition to direct skin- and coat-related outcomes, evaluating fecal microbial composition may provide complementary insight into the broader physiological responses to nutritional intervention. In the present study, fecal microbiota analysis was included as an exploratory dimension to assess whether SH and ESM supplementation were accompanied by shifts in microbial community structure in healthy cats.

Against this backdrop, a significant knowledge gap remains. Existing feline studies have mainly focused on allergic skin disease, single nutritional outcomes, or broad microbiome descriptions, whereas the potential benefits of dietary SH, ESM, or their combination on feline skin and coat health have not been systematically compared. In particular, it is unknown whether supplementation with SH alone, ESM alone, or a 1:1 mixture of the two differentially affects hair microstructure, skin barrier function, systemic antioxidant and inflammatory status, nutrient deposition in hair, and fecal microbial composition in healthy cats. Therefore, the present study was designed to evaluate and compare the effects of dietary SH, ESM, and SH + ESM in healthy Ragdoll cats, using an integrated evaluation system that includes hair microstructure, coat sensory traits, skin barrier function, hematological and serum biomarkers, hair trace elements and amino acids, and fecal microbiota.

## 2 Materials and methods

### 2.1 Animals and dietary treatments

The animal experimental protocol was reviewed and approved by the Laboratory Animal Ethics Committee of China Agricultural University (Approval No.: AW80215202-5-07). All procedures complied with relevant international guidelines for animal experimentation and Chinese regulations on laboratory animal management. Every effort was made to ensure animal welfare and to minimize discomfort. The SH product used in the present study had a molecular weight distribution of 200–600 kDa. According to the manufacturer’s specifications, the ESM product contained 6.8% collagen and 0.16% chondroitin sulfate. Twenty-four 6-month-old Ragdoll kittens were randomly assigned to four groups (*n* = 6 per group) and housed individually in cat cages (3 m × 1 m × 2 m). The control group (CON) received a basal diet, while the treatment groups received the basal diet supplemented with 250 mg/kg SH, 250 mg/kg ESM, or a combination of 125 mg/kg SH plus 125 mg/kg ESM (SH + ESM). Routine immunization and deworming were completed prior to the commencement of the study. Litter boxes were changed daily, and the enclosures were cleaned and maintained to ensure hygienic conditions. Throughout the trial, cats were monitored daily for general appetite, clinical condition, and any adverse events. All cats were fed a nutritionally complete diet formulated to meet the standards of the American Association of Feed Control Officials (AAFCO) and the National Research Council (NRC), provided twice daily, with ad libitum access to water.

Following a 7-day acclimation period, the designated amounts of SH and/or ESM were weighed and thoroughly mixed with the basal diet immediately before each daily feeding. Supplementation was administered for 56 consecutive days. Body weight was recorded on days 0, 14, 28, 42, and 56 (Table 1).

**Table 1:** Ingredient composition and nutrient profile of the basal diet.

Ingredients	Content (%)
Fresh chicken	25.00
Chicken meat meal	22.00
Corn	13.00
Brown rice	13.00
Wholemeal	10.00
Chicken oil	6.00
Beetroot meal	4.00
Alfalfa pellets	2.50
Vitamins and minerals premix^a^	1.70
Butter	0.80
Whole egg powder	1.00
Soya lecithin	0.30
Taurine	0.10
Spirulina powder	0.10
Plantago	0.15
Yucca extract	0.10
Beer yeast	0.25
Total	100.00
Nutrient content^b^	
DM	89.50
CP	30.20
EE	16.20
Ash	8.50
WSC	1.10

^a^ Provided per kilogram diet: vitamin A, 10,000.00 IU; vitamin D3, 800.00 IU; vitamin E, 120.00 mg; vitamin K3, 1.00 mg; vitamin B1, 12.00 mg; vitamin B2, 6.00 mg; vitamin B6, 5.00 mg; vitamin B12, 0.05 mg; nicotinic acid, 50.00 mg; calcium pantothenate, 20.00 mg; D-biotin, 0.20 mg; folic acid, 0.80 mg; Choline chloride, 2000 mg; Fe, 80.00 mg; Cu, 10.00 mg; Mn, 7.50 mg; Zn, 80.00 mg; I, 1.50 mg; Se, 0.20 mg.

^b^ DM, dry matter; CP, crude protein; EE, ether extract; WSC, water soluble carbohydrates.

### 2.2 Sample collection

On day 56, approximately 2 mL of blood was collected from the forelimb vein of each cat; one aliquot was transferred into an EDTA-containing tube for routine hematological analysis, and the remaining aliquot was placed in a sterile tube, allowed to clot at room temperature for 30 min, and centrifuged at 3,500 rpm for 10 min at 4 °C, after which the serum was harvested and stored at −80 °C for subsequent biochemical analyses. Fresh fecal samples were collected from sterile filter paper placed in each cat’s litter box, and approximately 5 g of feces was transferred into a sterile 50 mL tube using sterile forceps while minimizing environmental contamination; samples were immediately frozen and stored at −80 °C until microbiome analysis. In addition, hair samples were collected from the dorsal region of each cat using sterile scissors, consistently from the same site in all cats, placed in sterile containers, sealed, air-dried, and stored for subsequent scanning electron microscopy (SEM) analysis.

### 2.3 Hair parameter analysis

To evaluate hair microstructure and biochemical composition, hair samples were subjected to scanning electron microscopy (SEM) and free amino acid (FAA) profiling, with procedures adapted from published methods with minor modifications. For SEM observation, representative hair fibers were randomly selected from the same dorsal site of each cat. Hair fibers were gently rinsed with deionized water to remove surface debris and sebum residues, briefly washed with 70% ethanol, and air-dried. Dried fibers were mounted on SEM stubs using conductive carbon tape and sputter-coated with gold (or gold–palladium) to enhance conductivity. Samples were then examined using a scanning electron microscope (SU8100, Hitachi, Tokyo, Japan). Images were acquired under standardized settings and magnifications, with particular attention to cuticle scale morphology, including scale arrangement, edge integrity, lifting, and surface damage, to allow consistent comparisons among groups.

For free amino acid (FAA) profiling analysis, approximately 50 mg of hair was weighed into sterile tubes, immediately placed on dry ice, and stored at low temperature until analysis. Prior to UHPLC measurement, FAAs were extracted using an appropriate solvent under vigorous mixing and/or ultrasonication, followed by centrifugation; the supernatant was collected and filtered through a 0.22 μm membrane. FAA composition was determined by ultra-high-performance liquid chromatography (UHPLC; APExBIO Technology LLC, Shanghai, China) and quantified using external calibration with amino acid standards. Quality control samples were analyzed within each batch to ensure analytical stability and data comparability. For trace element analysis, the samples were washed sequentially with acetone, ultrapure water, and acetone, dried at 60 °C to constant weight, and homogenized. Approximately 0.100 g of dried hair was digested with 5 mL nitric acid and 1 mL hydrogen peroxide in a microwave digestion system. After digestion, the solution was diluted to 25 mL with ultrapure water, and Zn, Fe, Cu, and Mn concentrations were determined by inductively coupled plasma mass spectrometry (ICP-MS; Agilent 7,900, Agilent Technologies, Tokyo, Japan).

### 2.4 Fur quality assessment

A panel of six trained evaluators assessed coat condition according to standardized criteria. Based on the fur condition scoring system, coat quality was evaluated in a double-blind manner using three sensory indices: hair gloss, hair compliance, and skin elasticity. For hair gloss, scores ranged from 1 (dull) to 5 (bright and glossy); for hair compliance, scores ranged from 1 (matted) to 5 (smooth and compliant); and for skin elasticity, scores ranged from 1 (no recoil) to 5 (immediate recovery). In addition, coat color differences were measured using a colorimeter (NR20XE, Foshan, China) at the mid-dorsal region. The mid-dorsal region was defined as the point where the highest point of the scapula intersects with the dorsal midline. Color values were expressed using the CIE L*, a*, and b* coordinates, representing lightness (L*), redness/greenness (a*), and yellowness/blueness (b*), respectively.

### 2.5 Skin barrier assessment

TEWL was measured using a closed-chamber instrument (GPSkin Evolution®, GPOWER Inc., Seoul, South Korea) to evaluate skin barrier function and integrity, following the method described by Silva Carvalho et al. (14). The dorsal skin was selected as the measurement site. Before assessment, hair in the target area was carefully clipped using a pet hair trimmer to avoid skin injury, and no liquids or gels were applied throughout the procedure. TEWL was measured three consecutive times at each site, and the mean value was used for subsequent analysis. All measurements were performed by the same operator under controlled environmental conditions (22–25 °C and 60–70% relative humidity), with efforts made to ensure measurements were taken at the same location each time. Assessments were conducted on days 28 and 56. In parallel, skin images were acquired using a skin and hair analyzer (hairMetrix® Vidix, Canfield Scientific, Inc., Fairfield, NJ, USA) to evaluate dorsal skin surface texture, hair follicle condition, and skin moisture and sebum secretion. Image acquisition and analysis were performed at the same dorsal site, under the same environmental conditions, and at the same time points as TEWL measurement to ensure consistency and comparability of the results.

### 2.6 Hematology and serum antioxidant, trace element and vitamin analyses

After blood collection, routine hematological parameters were determined using an automated hematology analyzer (Contec, Qinhuangdao, Hebei, China). Serum antioxidant capacity and oxidative damage-related biomarkers were measured using enzyme-linked immunosorbent assay (ELISA) kits (Shanghai Enzyme-linked Biotechnology Co., Ltd., Shanghai, China) according to the manufacturers’ instructions, including superoxide dismutase (SOD), malondialdehyde (MDA), glutathione peroxidase (GSH-Px), and catalase (CAT). In addition, interleukin-31 (IL-31), an inflammation/pruritus-related cytokine, was quantified using the same ELISA kit platform. Total antioxidant capacity (T-AOC) was assessed using a micro-method colorimetric assay. Serum trace elements, including zinc (Zn) and copper (Cu), were determined by inductively coupled plasma mass spectrometry (ICP-MS; NexION 300D, PerkinElmer, USA). Fat-soluble antioxidant vitamins A (VA) and vitamin E (VE) were quantified by high-performance liquid chromatography (HPLC; 1,200 Series, Agilent Technologies, USA). All measurements were performed within the same analytical batch, and quality control samples were included to ensure accuracy and reproducibility.

### 2.7 16S rRNA gene sequencing and microbial bioinformatics analysis

Microbial genomic DNA was extracted from fecal samples using the E. Z. N. A.® Soil DNA Kit (Omega Bio-tek, Norcross, GA, USA) according to the manufacturer’s instructions. DNA integrity was evaluated by 1% agarose gel electrophoresis, and DNA concentration and purity were assessed using a NanoDrop™ 2000 spectrophotometer (Thermo Scientific, Waltham, MA, USA). Samples were considered suitable for downstream analyses when the A260/280 ratio was > 1.8 and the A260/230 ratio was > 2.0. The V3–V4 hypervariable region of the bacterial 16S rRNA gene was amplified by PCR using primers 341F (5′-CCTAYGGGRBGCASCAG-3′) and 806R (5′-GGACTACNNGGGGTATCTAAT-3′). PCR amplicons were pooled and purified using the AxyPrep DNA Gel Extraction Kit (Axygen Biosciences, Union City, CA, USA), and the purified products were quantified using a Quantus™ fluorometer (Promega, Madison, WI, USA). Sequencing libraries were constructed using the NEXTflex™ Rapid DNA-Seq Library Prep Kit (Bioo Scientific, Austin, TX, USA) following the manufacturer’s protocols, and paired-end sequencing (2 × 250 bp) was performed on an Illumina NovaSeq platform (Illumina, San Diego, CA, USA). Raw reads were demultiplexed and quality-filtered using fastp (v0.20.0), and paired-end reads were merged using FLASH (v1.2.7). High-quality sequences were clustered into operational taxonomic units (OTUs) at 97% similarity using UPARSE (v7.1), with chimeric sequences detected and removed during the clustering process. An OTU table was generated, and taxonomic assignment was performed using the Ribosomal Database Project (RDP) classifier (v2.13) against the Silva 138 database. Alpha diversity indices, including ACE, Chao1, Shannon, and Simpson, were calculated in QIIME 2 (v2020.6) based on the OTU table to evaluate within-sample richness and diversity. Beta diversity was assessed using Bray–Curtis dissimilarity, and principal coordinate analysis (PCoA) was performed in QIIME 2 to visualize differences in community structure among groups. Relative abundances of bacterial taxa were summarized across taxonomic levels, with genus-level profiles used for downstream comparisons. For statistical analysis, differences in dominant taxa among groups were evaluated using the Wilcoxon rank-sum test (two-sided), as appropriate. A *p* value < 0.05 was considered statistically significant.

### 2.8 Statistical analyses

Statistical analyses were performed using IBM SPSS Statistics (version 24.0; IBM Corp., Armonk, NY, USA). For most parameters (e.g., hair parameters, fur quality scores, TEWL, hematology, serum biomarkers, trace elements, and vitamins), differences among groups were evaluated by one-way analysis of variance (ANOVA) followed by Tukey’s *post hoc* multiple-comparison test. For microbial relative abundances (at genus and higher taxonomic levels), differences between groups were assessed using the two-sided Wilcoxon rank-sum test. All tests were two-tailed, and *p* < 0.05 was considered statistically significant. Figures were generated using GraphPad Prism (version 10.1.2; GraphPad Software, San Diego, CA, USA).

## 3 Results

### 3.1 Experimental design and body weight changes

**Figure 1:**
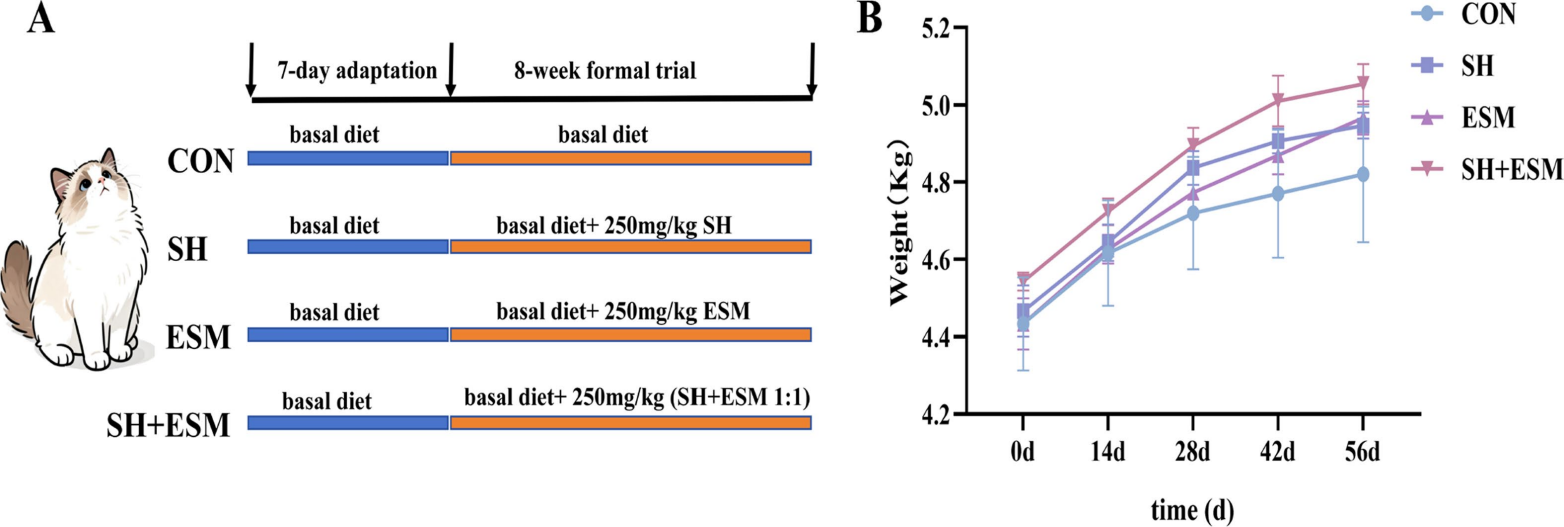
Study design and body weight changes. **(A)** Experimental timeline and dietary treatments. **(B)** Body weight measured on days 0, 14, 28, 42, and 56 among different groups. The values are expressed as means ± sem, *n* = 6.

Figure 1 shows the study design (A) and body weight changes (B). Following a 7-day adaptation, cats received the experimental diets for 8 weeks. Over the 56-day period, body weight increased progressively in all groups, reflecting normal growth. Although the SH + ESM group tended to have slightly higher body weight at most time points, no significant differences in weight gain were observed among groups, indicating that the supplements did not affect body weight under the conditions of this study.

### 3.2 Hair microstructure analysis

**Figure 2:**
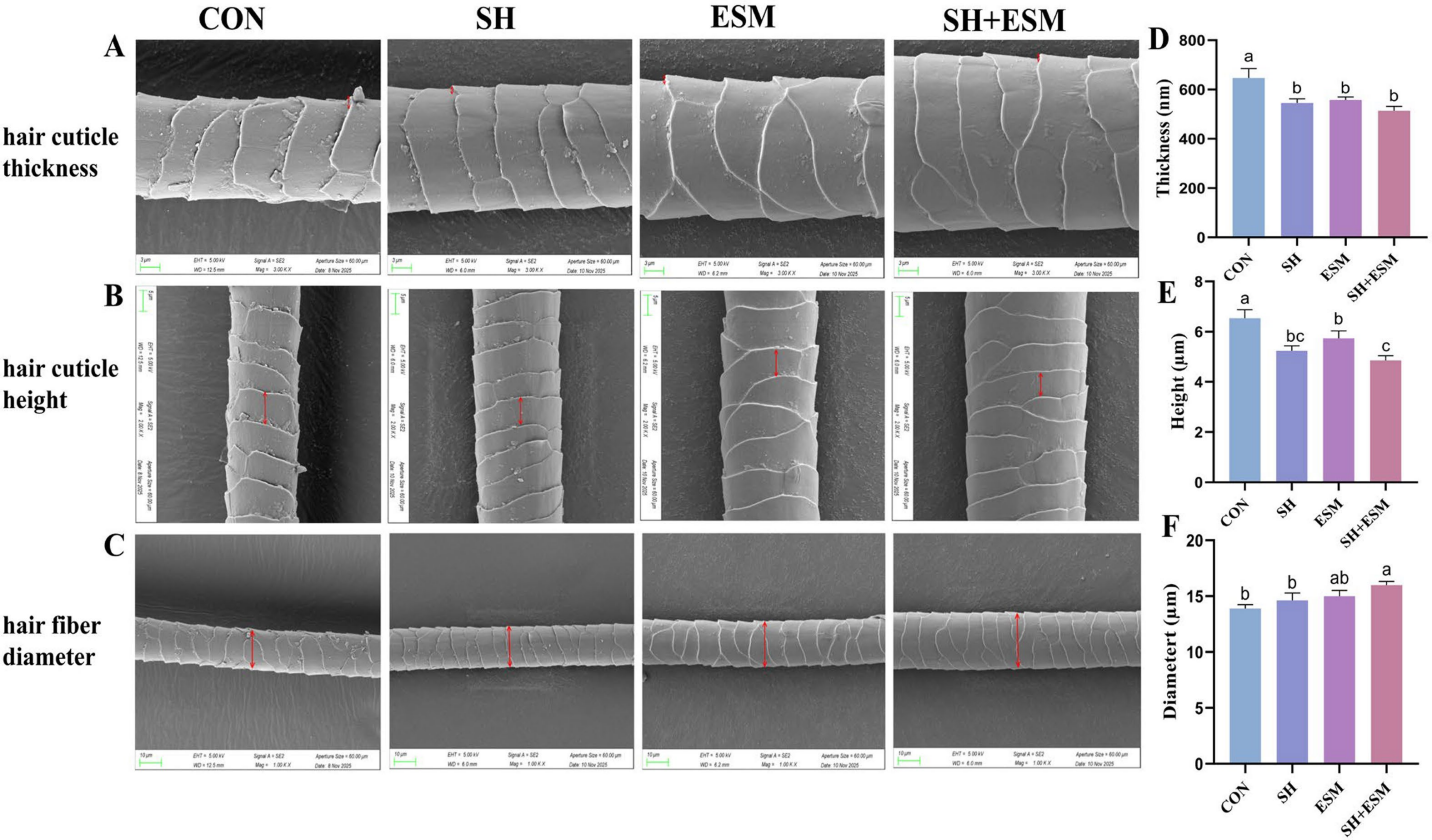
Effects of SH, ESM, and SH + ESM on cat hair structural characteristics. Representative scanning electron microscopy images of hair from the CON, SH, ESM, and SH + ESM groups showing **(A)** hair cuticle thickness at 3,000 × magnification, scale bar = 3 μm; **(B)** hair cuticle height at 2,000 × magnification, scale bar = 5 μm; and **(C)** hair fiber diameter at 1,000 × magnification, scale bar = 10 μm. All images within the same row were acquired at the same magnification. **(D–F)** Quantitative comparisons of hair cuticle thickness, height, and diameter among CON, SH, ESM, and SH + ESM, respectively. Data are expressed as means ± sem (*n* = 6), and different letters indicate significant differences among groups (*p* < 0.05).

Figure 2 presents representative SEM images (A-C) and quantitative hair microstructure parameters (D-F). All supplemented groups (SH, ESM, and SH + ESM) showed a more uniform cuticle surface with better scale alignment than the CON group. Compared with CON, cuticle thickness was significantly reduced in all treated groups (*p* < 0.05; Figure 2D), with no differences among them. The SH + ESM group exhibited the lowest cuticle height (*p* < 0.05; Figure 2E) and the largest hair fiber diameter compared with all other groups (*p* < 0.05; Figure 2F). These findings indicate that supplementation, particularly the combination of SH and ESM, is associated with smoother, more compact cuticle morphology and increased fiber diameter, suggesting improved hair structural integrity.

### 3.3 Coat sensory scores and colorimetric parameters

Dietary supplementation with SH, ESM, or their combination differentially affected coat quality parameters (Table 2). For hair gloss, only the SH + ESM group showed a significant improvement compared with CON (*p* < 0.05), whereas SH or ESM alone had no effect. Hair compliance was significantly increased by ESM and SH + ESM, and skin elasticity by SH and SH + ESM groups compared with CON (*p* < 0.05). Coat brightness (L*) was increased in all supplemented groups relative to CON (*p* < 0.05), with SH and ESM showing comparable effects (no significant difference between them), while the SH + ESM combination yielded the highest brightness compared with all other groups (*p* < 0.05). Redness (a*) was elevated only in the SH + ESM group compared with CON (*p* < 0.05), whereas yellowness (b*) remained unchanged. Taken together, these findings demonstrate that combined SH and ESM supplementation exerts superior benefits on feline coat visual appearance, tactile quality, and skin elasticity compared with either ingredient alone.

**Table 2:** Effects of SH, ESM, and SH + ESM on coat sensory scores and colorimetric parameters in cats.

Item	CON	SH	ESM	SH + ESM	*p-*Value
hair gloss	2.83 ± 0.41^bc^	3.67 ± 0.52^b^	3.58 ± 0.49^b^	4.67 ± 0.52^a^	<0.010
hair compliance	3.33 ± 0.52^b^	3.83 ± 0.41^ab^	4.50 ± 0.55^a^	4.58 ± 0.38^a^	<0.010
Skin elasticity	2.58 ± 0.49^b^	4.00 ± 0.32^a^	3.50 ± 0.63^ab^	4.50 ± 0.55^a^	<0.010
Brightness (L*)	63.93 ± 2.82^d^	66.70 ± 1.35^bc^	67.46 ± 0.85^b^	71.22 ± 1.30^a^	<0.010
Redness (a*)	0.47 ± 0.06^b^	0.54 ± 0.10^ab^	0.58 ± 0.04^ab^	0.68 ± 0.06^a^	0.026
Yellowness (b*)	7.69 ± 0.10	8.07 ± 0.20	8.46 ± 0.83	8.79 ± 0.72	0.168

Data are expressed as means ± sem (*n* = 6), and different letters indicate significant differences among groups (*p* < 0.05).

### 3.4 Skin condition and barrier function

**Figure 3:**
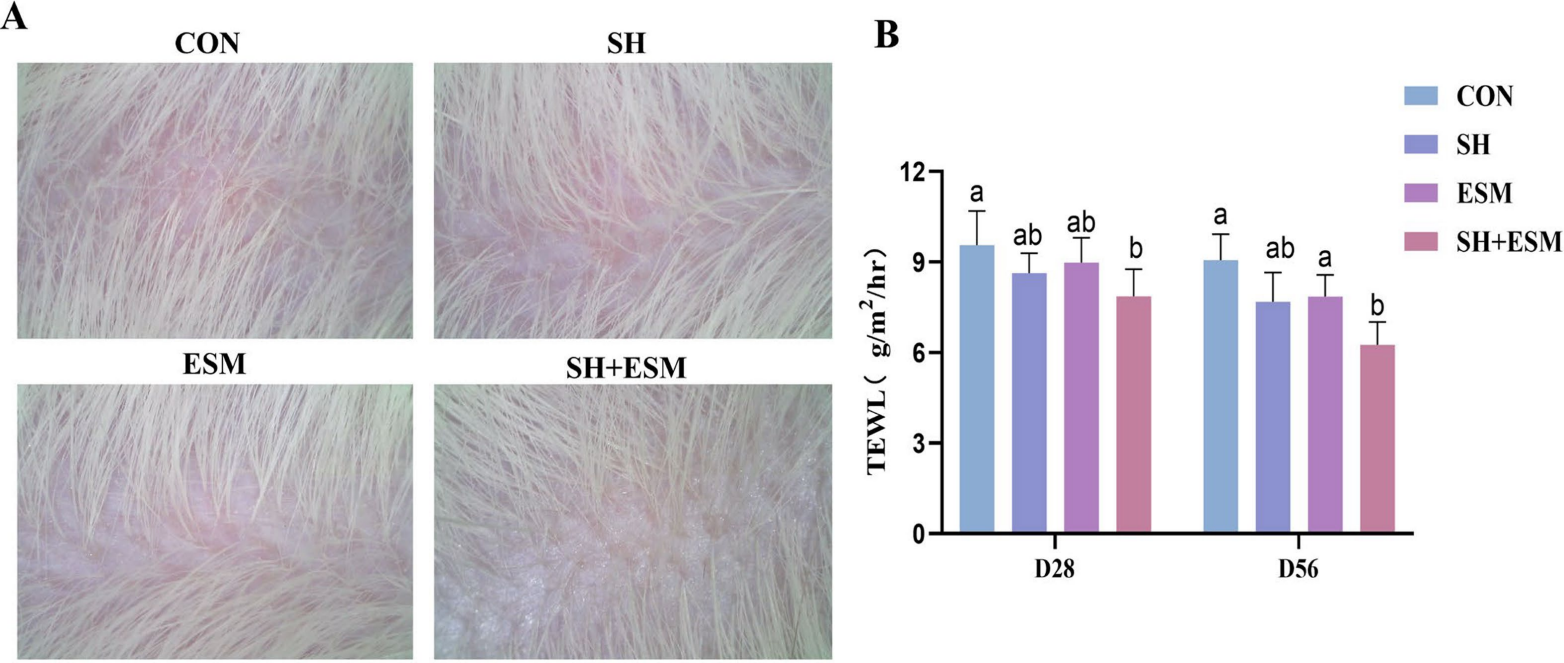
Effects of SH, ESM, and SH + ESM on skin appearance and barrier function. **(A)** Representative dorsal skin images acquired using a skin and hair analyzer on day 56. **(B)** Transepidermal water loss (TEWL) measured on days 28 and 56. Data are expressed as means ± sem (*n* = 6), and different letters indicate significant differences among groups (*p* < 0.05).

Skin surface characteristics and TEWL are presented in Figures 3A,B. Qualitative imaging on day 56 showed that the CON group had a dry, coarse surface with follicular irregularities and fine flakes. In contrast, SH and ESM groups exhibited improved surface smoothness, follicular definition, and reduced flaking, with ESM additionally promoting a more compact microstructure. The SH + ESM combination produced the most pronounced enhancement, characterized by the smoothest surface, best-defined follicular openings, and minimal flaking. TEWL measurements (Figure 3B) confirmed these observations. At day 28, SH + ESM showed significantly lower TEWL than CON (*p* < 0.05), while SH and ESM were intermediate. By day 56, TEWL remained lowest in SH + ESM and was significantly lower than in CON and ESM (*p* < 0.05), with SH again showing an intermediate value. Collectively, combined SH + ESM supplementation most consistently improved skin barrier function, as evidenced by both skin imaging and reduced TEWL.

### 3.5 Hair trace elements and amino acid profiles

Hair Zn content was higher ESM and SH + ESM groups than the CON group (*p* < 0.05), while hair Cu, Fe, and Mn levels did not differ significantly among different groups. Regarding hair free amino acid profiles, multiple amino acids displayed significant treatment-associated increases. Compared with the CON group, the SH, ESM, and SH + ESM groups showed significantly higher levels of alanine (Ala), valine (Val), methionine (Met), leucine (Leu), and histidine (His) (*p* < 0.05). Compared with the control group, the SH + ESM group showed significantly higher levels of lysine (Lys), proline (Pro), arginine (Arg), and cystine/cysteine-related content (Cys) (*p* < 0.05). The remaining amino acids, including Ile, Thr, Ser, Glu, Gly, Tyr, Phe, and Asp., showed no significant differences among groups or only non-significant trends. Collectively, these results indicate that dietary SH + ESM supplementation influenced the deposition of Zn and several key amino acids in hair. Among the treatment groups, the SH + ESM group showed more evident increases in nutrients closely associated with hair structural proteins (Table 3).

**Table 3:** Effects of SH, ESM, and SH + ESM on the trace element and amino acid contents in cat hair.

Item	CON	SH	ESM	SH + ESM	*p-*value
Zn (mg/kg)	152.66 ± 3.51^d^	159.33 ± 2.52^cd^	165.67 ± 3.51^bc^	174.67 ± 11.37^a^	0.036
Cu (mg/kg)	9.54 ± 1.51	9.78 ± 0.97	8.93 ± 0.78	10.23 ± 0.70	0.188
Fe (mg/kg)	35.03 ± 5.27	38.53 ± 4.75	30.10 ± 7.70	28.40 ± 6.99	0.101
Mn (mg/kg)	1.13 ± 0.11	1.24 ± 0.13	1.21 ± 0.26	1.34 ± 0.16	0.203
Ile (%)	0.39 ± 0.07	0.47 ± 0.05	0.45 ± 0.08	0.51 ± 0.06	0.079
Thr (%)	5.56 ± 1.38	5.89 ± 0.27	5.89 ± 0.07	5.77 ± 0.21	0.610
Ser (%)	8.90 ± 0.19	8.44 ± 0.31	8.80 ± 0.28	8.65 ± 0.39	0.117
Glu (%)	13.44 ± 0.39	13.11 ± 0.52	13.66 ± 0.21	13.41 ± 0.28	0.123
Gly (%)	5.75 ± 0.19	5.55 ± 0.12	5.69 ± 0.02	5.73 ± 0.10	0.089
Ala (%)	3.52 ± 0.12^b^	3.73 ± 0.07^a^	3.79 ± 0.03^a^	3.78 ± 0.13^a^	0.008
Val (%)	4.77 ± 0.19^b^	5.08 ± 0.07^a^	5.08 ± 0.04^a^	5.09 ± 0.15^a^	0.015
Met (%)	2.66 ± 0.08^b^	2.79 ± 0.07^a^	2.81 ± 0.01^a^	2.82 ± 0.04^a^	0.007
Leu (%)	6.37 ± 0.13^b^	6.61 ± 0.11^a^	6.79 ± 0.02^a^	6.70 ± 0.14^a^	0.002
Tyr (%)	3.81 ± 0.14	3.64 ± 0.16	3.86 ± 0.13	3.84 ± 0.11	0.107
Phe (%)	7.90 ± 0.74	8.47 ± 0.30	8.59 ± 0.05	8.43 ± 0.25	0.396
Lys (%)	4.26 ± 0.19^b^	4.45 ± 0.15^ab^	4.57 ± 0.23^ab^	4.61 ± 0.12^a^	0.039
His (%)	1.41 ± 0.05^b^	1.52 ± 0.03^a^	1.48 ± 0.04^ab^	1.51 ± 0.02^a^	0.006
Pro (%)	6.60 ± 0.22^b^	6.69 ± 0.26^ab^	6.74 ± 0.10^ab^	7.05 ± 0.21^a^	0.028
Arg (%)	2.87 ± 0.12^b^	3.01 ± 0.03^ab^	3.09 ± 0.03^a^	3.11 ± 0.10^a^	0.008
Asp (%)	7.35 ± 0.32	8.08 ± 0.54	7.44 ± 0.72	8.31 ± 0.11	0.052
Cys (%)	6.28 ± 0.16^b^	6.47 ± 0.19^ab^	6.43 ± 0.12^ab^	6.59 ± 0.05^a^	0.026

Data are expressed as means ± sem (*n* = 6), and different letters indicate significant differences among groups (*p* < 0.05).

### 3.6 Blood routine indicators

As shown in Table 4, the SH + ESM group exhibited a significant decrease in granulocyte ratio compared with the CON group (*p* < 0.05). Regarding granulocyte count, both the ESM and SH + ESM groups showed significant reductions relative to the CON group (*p* < 0.05). In contrast, total red blood cells, hemoglobin, and hemoglobin content were significantly higher in the SH + ESM group than in the ESM group (*p* < 0.05), with no other significant differences among groups for these parameters. No significant differences were observed for other hematological parameters across groups.

**Table 4:** Effects of SH, ESM, and SH + ESM in the blood routine parameters of cats.

Item	CON	SH	ESM	SH + ESM	Normal reference range	*p-*value
Total White Blood Cells (10^9^/L)	15.03 ± 4.89	14.20 ± 1.50	15.30 ± 5.11	13.87 ± 1.6	5.5–19.5	0.648
Lymphocyte Ratio (%)	42.50 ± 2.33	43.37 ± 5.45	42.67 ± 2.44	43.93 ± 2.70	12.0–45.0	0.627
Intermediate Cell Ratio (%)	6.13 ± 1.22	6.97 ± 1.59	6.83 ± 0.87	6.67 ± 1.25	2.0–9.0	0.442
Granulocyte Ratio (%)	47.37 ± 5.29^a^	36.00 ± 2.74^ab^	43.90 ± 15.7^ab^	26.07 ± 9.03^b^	35.0–85.0	0.026
Lymphocytes (10^9^/L)	6.03 ± 2.15	6.20 ± 1.22	5.13 ± 1.10	5.33 ± 0.76	0.8–7.0	0.379
Intermediate Cells (10^9^/L)	1.03 ± 0.38	1.18 ± 0.24	1.00 ± 0.26	0.93 ± 0.25	0.1–1.9	0.331
Granulocytes (10^9^/L)	6.97 ± 1.71^a^	5.07 ± 0.38^ab^	6.17 ± 0.15^b^	3.60 ± 1.31^b^	2.1–15.0	0.006
Total Red Blood Cells (10^12^/L)	8.36 ± 0.95^ab^	7.82 ± 0.06^ab^	7.32 ± 0.42^b^	9.18 ± 1.51^a^	4.60–10.00	0.038
Hemoglobin (g/L)	133.67 ± 20.21^ab^	121.67 ± 1.15^ab^	114 ± 13.11^b^	157.67 ± 36.14^a^	93–153	0.039
Hematocrit (%)	33.33 ± 4.59	30.40 ± 0.56	36.53 ± 5.07	29.33 ± 3.39	28.0–49.0	0.050
Mean Corpuscular Volume (fL)	39.87 ± 1.07	39.07 ± 0.76	40.00 ± 1.74	41.20 ± 3.01	39.0–52.0	0.198
Hemoglobin Content (pg)	15.97 ± 0.70^ab^	15.53 ± 0.25^ab^	15.73 ± 1.21^b^	17.70 ± 1.57^a^	13.0–21.0	0.037
Hemoglobin Concentration (g/L)	373.67 ± 15.04	378.67 ± 14.19	367.67 ± 25.58	375.33 ± 17.79	300–380	0.492
Red Cell Distribution Width SD (fL)	30.43 ± 2.20	29.80 ± 1.95	31.57 ± 3.23	33.47 ± 5.50	47.0–62.7	0.237
Red Cell Distribution Width CV (%)	17.2 ± 1.91	16.50 ± 1.50	16.43 ± 1.01	16.77 ± 1.99	14.0–18.0	0.584
Total Platelet Count (10^9^/L)	347.67 ± 157.00	274.67 ± 68.19	490.67 ± 58.19	322.33 ± 143.5	100–514	0.242
Mean Platelet Volume (fL)	10.43 ± 0.06	10.57 ± 0.45	10.77 ± 0.65	9.33 ± 1.72	5.0–11.8	0.100
Platelet Distribution Width (%)	9.2 ± 0.30	9.40 ± 0.46	8.60 ± 1.49	9.13 ± 1.10	0.1–30.0	0.340
Plateletcrit (%)	0.36 ± 0.17	0.29 ± 0.08	0.59 ± 0.49	0.36 ± 0.41	0.01–9.99	0.293
Platelet–Larger Cell ratio (%)	26.2 ± 7.45	23.17 ± 3.26	23.23 ± 5.61	20.13 ± 6.09	0.1–99.9	0.236

Data are expressed as means ± sem (*n* = 6), and different letters indicate significant differences among groups (*p* < 0.05).

### 3.7 Serum antioxidant status

**Figure 4:**
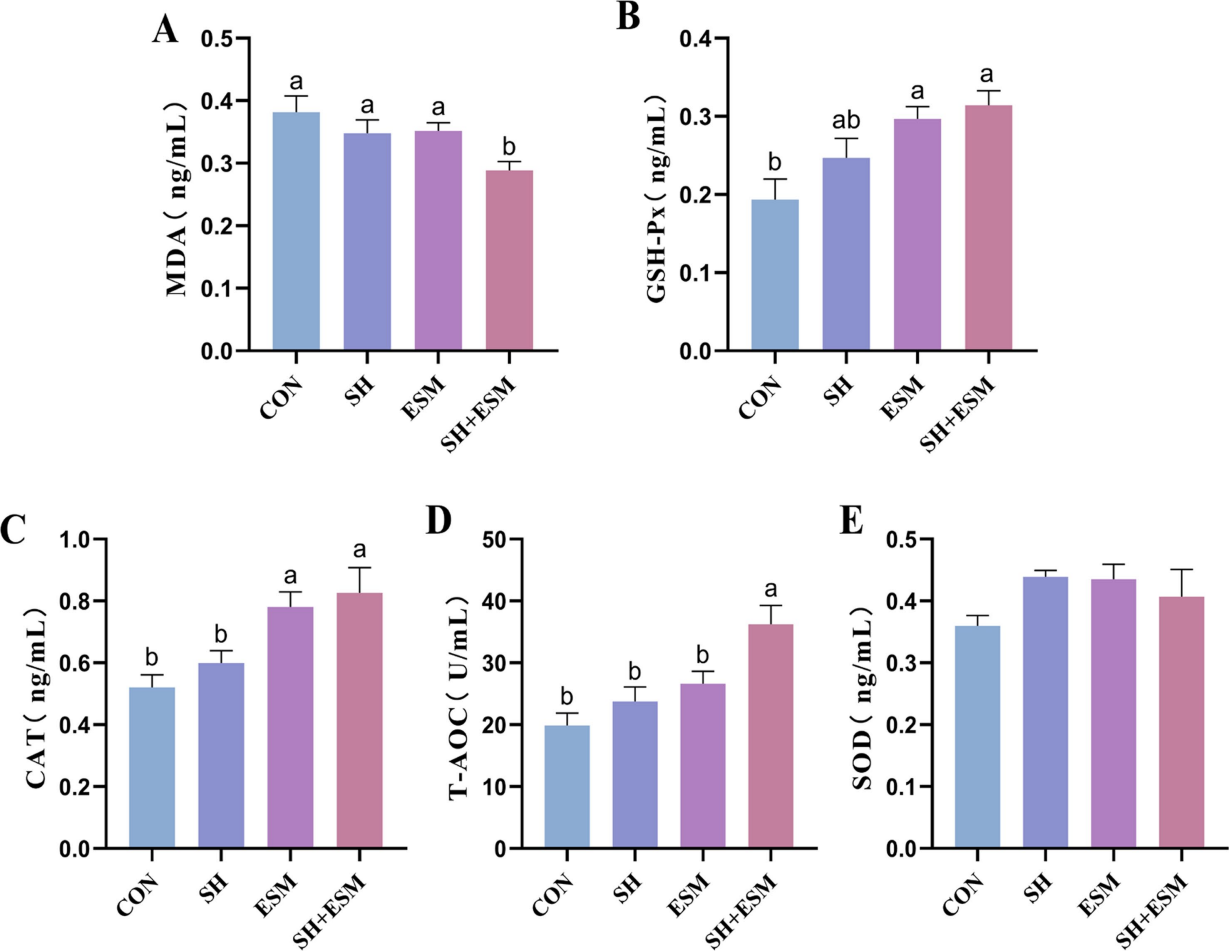
Effects of SH, ESM, and SH + ESM supplementation on serum oxidative stress and antioxidant indices in cats. **(A)** MDA. **(B)** GSH-Px. **(C)** CAT. **(D)** T-AOC. **(E)** SOD. Data are expressed as means ± sem (*n* = 6), and different letters indicate significant differences among groups (*p* < 0.05).

After 56 days of supplementation, the SH + ESM group showed a significantly lower serum MDA level than the CON, SH, and ESM groups (*p* < 0.05, Figure 4A). Antioxidant enzyme activities also increased: GSH-Px was significantly elevated in both the ESM and SH + ESM groups relative to CON (*p* < 0.05, Figure 4B), and CAT levels in the ESM and SH + ESM groups were significantly higher than those in CON and SH (*p* < 0.05, Figure 4C). Furthermore, T-AOC was significantly higher in the SH + ESM group than in all other groups (*p* < 0.05, Figure 4D). In contrast, SOD activity did not differ significantly among groups (*p* > 0.05, Figure 4E). Collectively, these findings indicate that combined SH + ESM supplementation improves systemic antioxidant status more effectively, as evidenced by reduced lipid peroxidation and enhanced antioxidant defense capacity.

### 3.8 Skin-related serum biomarkers

**Figure 5:**
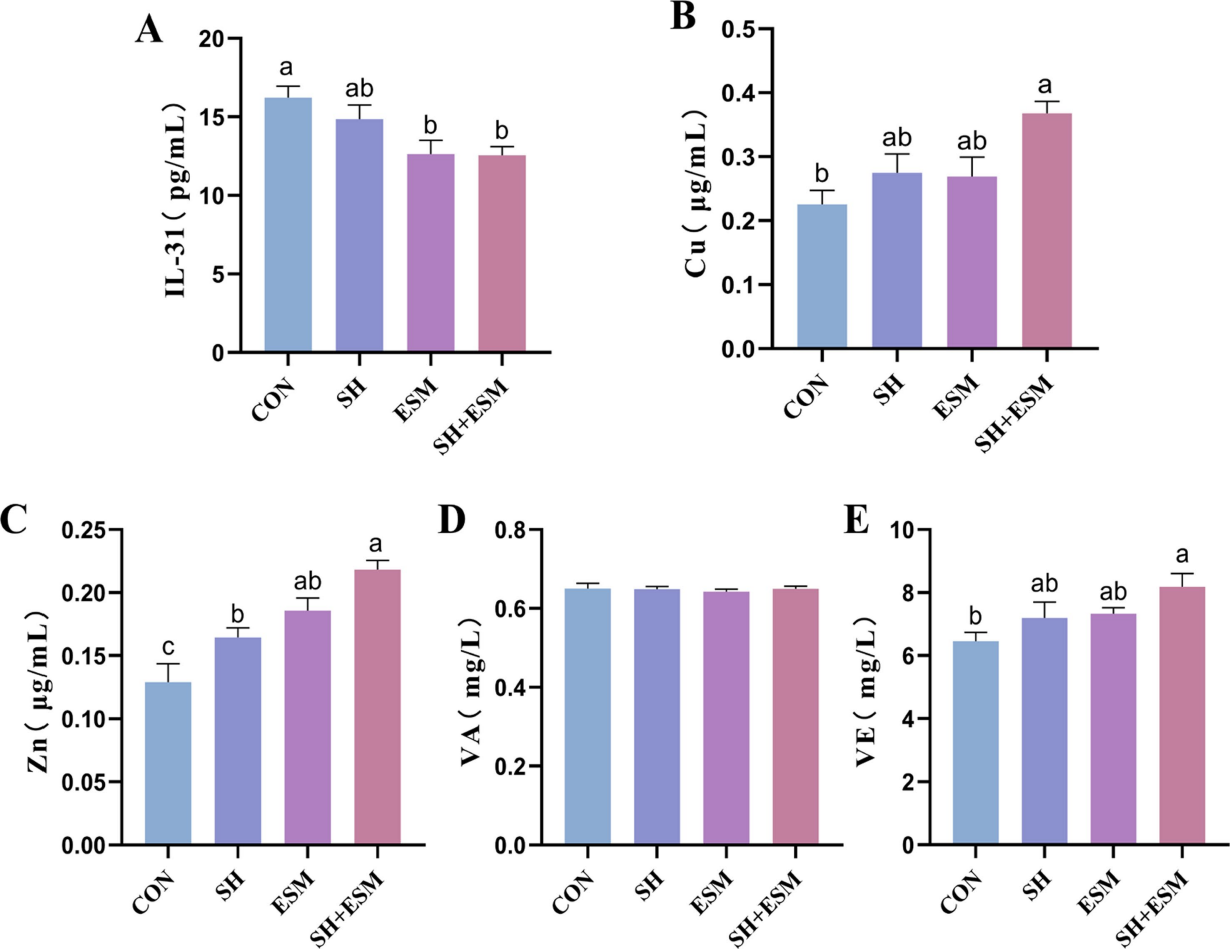
Effects of SH, ESM, and SH + ESM supplementation on skin-related serum biomarkers in cats. **(A)** IL-31. **(B)** Cu. **(C)** Zn. **(D)** Vit A. **(E)** Vit E. Data are expressed as means ± sem (*n* = 6), and different letters indicate significant differences among groups (*p* < 0.05).

After 56 days of dietary supplementation, serum IL-31 levels were significantly lower in the ESM and SH + ESM groups than in the CON group (*p* < 0.05, Figure 5A), whereas the SH group showed intermediate values. For serum trace elements, the SH + ESM group exhibited significantly higher Cu and Zn concentrations than the CON group (*p* < 0.05 for both; Figures 5B,C), with Zn reaching the highest level among all groups. Regarding fat-soluble antioxidant vitamins, serum Vit A levels did not differ significantly among groups (*p* > 0.05, Figure 5D). In contrast, serum Vit E was significantly increased in the SH + ESM group compared with the CON group (*p* < 0.05, Figure 5E), while numerical upward trends were observed in the SH and ESM groups. Collectively, these findings indicate that combined SH + ESM supplementation more effectively modulated skin health-related serum biomarkers in cats, as reflected by reduced IL-31 levels and increased vitamin E, Zn, and Cu concentrations. These changes may be associated with attenuation of IL-31-related inflammatory or pruritic responses, enhanced antioxidant protection, and improved availability of trace elements involved in skin barrier maintenance.

### 3.9 Fecal microbiota composition

**Figure 6:**
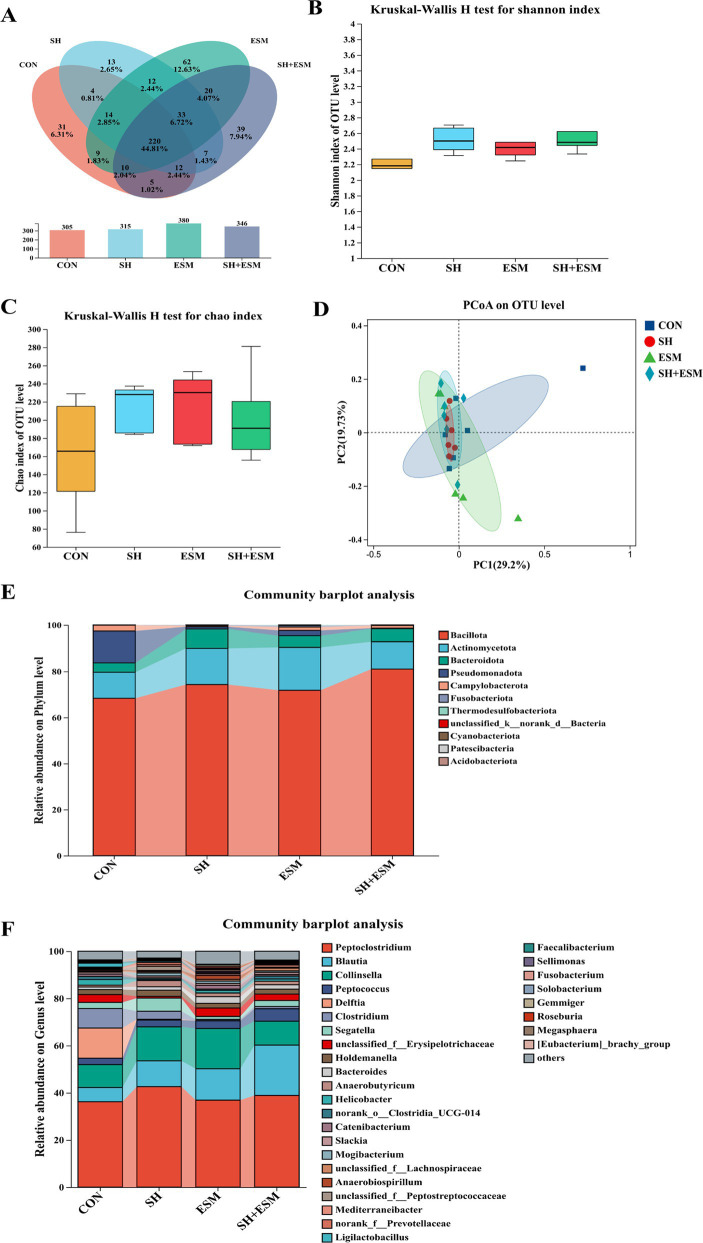
Effects of SH, ESM, and SH + ESM on the composition of fecal microbiota in cats **(A)** Venn **(B)** Shannon index **(C)** Chao index **(D)** principal coordinates analysis **(E,F)** bacterial composition at the phylum and genus levels, respectively. The values are expressed as means ± *sem*, *n* = 6 per group.

As shown in Figure 6A, the Venn diagram illustrates the distribution of OTUs among the four groups, revealing a core microbiota composed of 220 shared OTUs across all treatments. Each group also contained unique OTUs, with the ESM group exhibiting the highest number of unique OTUs (62) and the SH group the lowest (15). The total numbers of observed OTUs were 305, 315, 380, and 346 in the CON, SH, ESM, and SH + ESM groups, respectively. No significant differences were observed in the Shannon and Chao indices among the four groups (Figures 6B,C). As shown in Figure 6D, the PCoA plot based on OTU composition demonstrated a partial separation of microbial communities among the four groups, with PC1 and PC2 explaining 29.2 and 19.73% of the total variation, respectively. The ESM group showed relatively greater dispersion, suggesting a tendency toward higher inter-individual variability. In contrast, samples from the SH + ESM group clustered more closely, suggesting relatively lower within-group variation in fecal microbial community structure.

The compositional changes at the phylum level are shown in Figure 6E. The fecal microbiota of all groups was dominated by Bacillota, which accounted for approximately 68.6% in the CON group and increased to about 74.6, 72.1, and 81.0% in the SH, ESM, and SH + ESM groups, respectively. Actinomycetota also increased in the treatment groups, particularly in the SH and ESM groups, compared with the control. In contrast, the relative abundance of Pseudomonadota was markedly higher in the CON group and was substantially reduced after supplementation, especially in the SH and SH + ESM groups. Minor phyla, including Bacteroidota, Campylobacterota, Fusobacteriota, Thermodesulfobacteriota, and several low-abundance unclassified taxa, showed only limited variation among groups. At the genus level (Figure 6F), the microbiota in the CON group was primarily composed of *Peptoclostridium*, *Delftia*, *Collinsella*, *Blautia*, and *Clostridium*. Compared with the control, both SH and ESM supplementation increased the relative abundances of *Blautia* and *Collinsella*, while markedly reducing *Delftia*. Notably, the SH + ESM group exhibited the highest relative abundance of *Blautia* among all groups, together with a persistently high proportion of *Peptoclostridium* and a pronounced depletion of *Delftia*. Overall, these results indicate that although SH, ESM, and SH + ESM did not significantly alter alpha diversity, they clearly reshaped the fecal microbial composition of cats, with the SH + ESM treatment producing a more concentrated and structurally distinct community profile.

**Figure 7:**
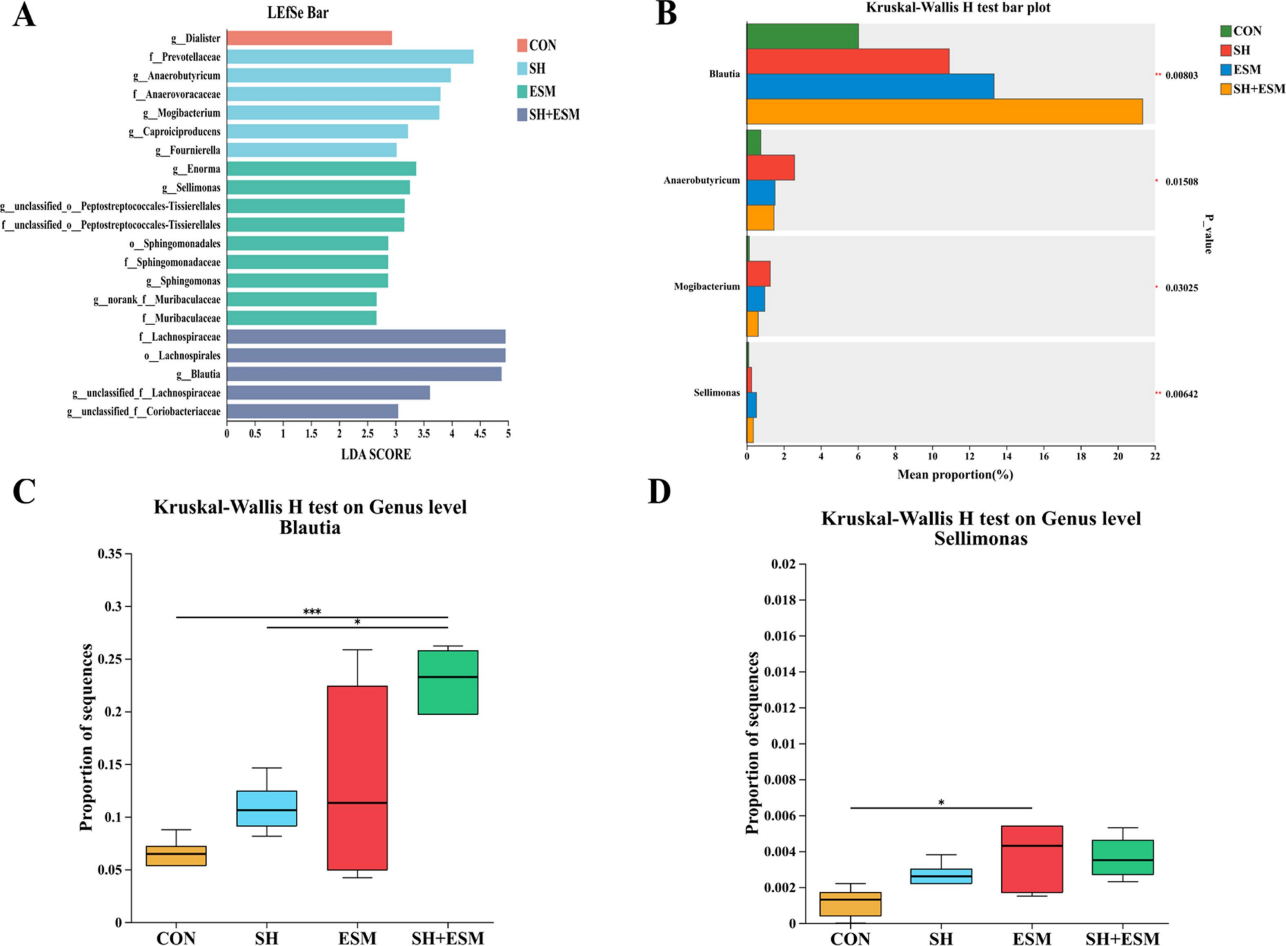
Effect of SH, ESM, and SH + ESM supplementation on feline gut microbiota composition **(A)** LEfSe (linear discriminant analysis effect size) bar plot; **(B)** Kruskal–Wallis H test bar plot; panels **(C,D)** depict the relative abundance of key microbial genera: **(C)** *Blautia*, **(D)** *Sellimonas*. Statistical significance: * *p* ≤ 0.05 and *** *p* ≤ 0.001. Data are represented as mean ± *sem* based on a sample size of *n* = 6 per group.

Microbial composition analyses using LEfSe (Linear discriminant analysis effect size) and Kruskal–Wallis H tests further revealed distinct taxonomic signatures among the four experimental groups (Figure 7). As shown in Figure 7A, LEfSe analysis identified multiple discriminatory taxa associated with each treatment. The CON group was characterized by the enrichment of *g__Dialister*, whereas the SH group showed preferential enrichment of *f__Prevotellaceae*, *g__Anaerobutyrium*, *f__Anaerovoracaceae*, *g__Mogibacterium*, *g__Caproiciproducens*, and *g__Fournierella*. In the ESM group, the discriminatory taxa mainly included *g__Enorma*, *g__Sellimonas*, unclassified Peptostreptococcales-Tissierellales taxa, *o__Sphingomonadales*, *f__Sphingomonadaceae*, *g__Sphingomonas*, and Muribaculaceae-related taxa. Notably, the SH + ESM group was distinguished by the enrichment of *o__Lachnospirales*, *f__Lachnospiraceae*, *g__Blautia*, unclassified *f__Lachnospiraceae*, and unclassified *f__Coriobacteriaceae*.

Genus-level differences were further assessed using the Kruskal–Wallis H test (Figure 7B), which identified *Blautia*, *Anaerobutyrium*, *Mogibacterium*, and *Sellimonas* as significantly altered taxa across groups, with corresponding *p* values of 0.00803, 0.01508, 0.03025, and 0.00642, respectively. Among these taxa, *Blautia* showed the most pronounced increase in the SH + ESM group, with a mean relative abundance exceeding those in the CON, SH, and ESM groups. As illustrated in Figure 7C, the relative abundance of *Blautia* was significantly higher in the SH + ESM group than in the CON group (*p* < 0.001) and SH group (*p* < 0.05), whereas the ESM group showed an intermediate but more variable distribution. In parallel, *Sellimonas* also differed significantly among groups (Figure 7D), with relatively higher proportions observed in the treatment groups, particularly in the ESM and SH + ESM groups, compared with the CON group. In addition, *Anaerobutyrium* and *Mogibacterium* were more abundant in the SH group than in the other groups, suggesting that SH supplementation selectively favored these taxa.

Collectively, these findings indicate that dietary SH, ESM, and combined SH + ESM supplementation were associated with taxonomic shifts in the feline fecal microbiota. Among the treatment groups, combined SH + ESM supplementation showed more evident increases in *g__Blautia* and *f__Lachnospiraceae*-associated taxa, suggesting that changes in fecal microbial composition may be associated with the observed improvements in skin- and coat-related outcomes.

### 3.10 Correlation between gut microbiota and serum indices

**Figure 8:**
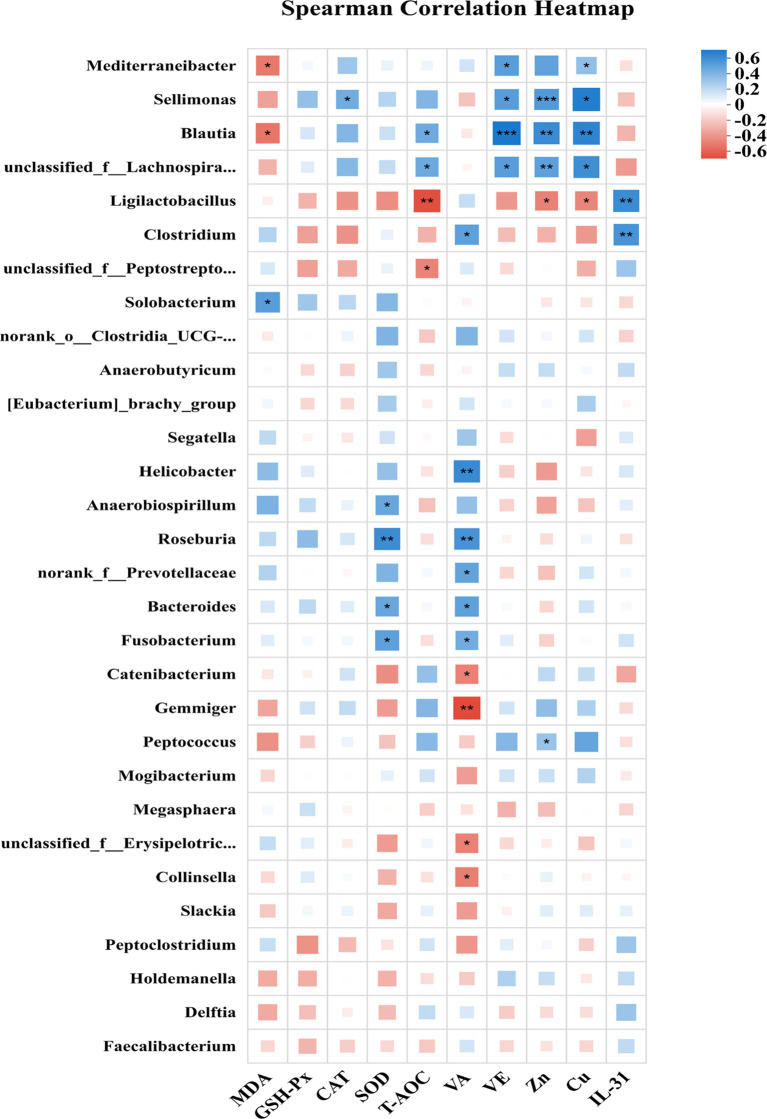
Spearman’s correlation heatmap depicting the associations between bacterial profiles and serum indices. Correlations are color-coded, with red representing positive associations and blue representing negative ones. The significance levels are * *p* ≤ 0.05, ** *p* ≤ 0.01, and *** *p* ≤ 0.001. Data are presented as mean ± sem, with a sample size of *n* = 6 per group.

Spearman correlation analysis was performed to explore the relationships between the 30 most abundant bacterial taxa and serum oxidative stress, antioxidant, micronutrient, and inflammatory indices (Figure 8). Overall, several genera showed clear associations with antioxidant-related parameters, trace elements, and IL-31, suggesting potential links between gut microbial composition and skin- or antioxidant-related host responses. Among the taxa positively associated with beneficial serum indices, *Blautia, Sellimonas*, and *unclassified_f__Lachnospiraceae* showed significant positive correlations with VE, Zn, and Cu, and in some cases with T-AOC, while being negatively associated with MDA. In particular, Blautia displayed a significant negative correlation with MDA and significant positive correlations with T-AOC, VE, Zn, and Cu, whereas Sellimonas was positively correlated with CAT, VE, Zn, and Cu. Similarly, Mediterraneibacter was negatively correlated with MDA and positively correlated with VE and IL-31.

By contrast, some taxa were associated with less favorable serum patterns. *Ligilactobacillus* showed significant negative correlations with T-AOC, Zn, and Cu, but a positive correlation with IL-31. In addition, *unclassified_f__Peptostreptococcaceae*, *Catenibacterium*, *Gemmiger*, *unclassified_f__Erysipelotrichaceae*, and *Collinsella* exhibited significant negative correlations with specific antioxidant- or micronutrient-related indices, particularly T-AOC or VA.

Several other genera were mainly associated with antioxidant enzyme activities. For example, *Roseburia*, *Bacteroides*, and *Fusobacterium* showed positive correlations with SOD and/or VA, whereas Helicobacter was positively associated with VA. In addition, Peptococcus showed a positive correlation with Zn, and Clostridium was positively correlated with VA and IL-31. Collectively, these findings indicate that specific bacterial taxa, particularly Blautia, Sellimonas, and related Lachnospiraceae members, were more closely associated with improved antioxidant and micronutrient profiles, whereas other taxa were linked to less favorable serum signatures.

## 4 Discussion

In the present study, dietary supplementation with SH and ESM, particularly their combination, improved multiple dimensions of skin and coat health in healthy Ragdoll cats without significantly affecting body weight. Specifically, the SH + ESM group showed the most consistent benefits, including improved hair-shaft morphology, increased hair fiber diameter, enhanced coat gloss and smoothness, improved skin elasticity and brightness, and reduced TEWL. These phenotypic improvements were accompanied by a more favorable systemic antioxidant profile, lower serum IL-31, higher concentrations of skin-related micronutrients, increased levels of several hair structural amino acids, and alterations in fecal microbial composition. Together, these findings suggest that the observed benefits were not attributable to changes in body weight, but rather to coordinated improvements in barrier integrity, oxidative balance, micronutrient status, and host–microbiota interactions. This interpretation is biologically plausible, although most available evidence comes from human clinical studies, oral SH supplementation has been reported to improve skin hydration and brightness in randomized clinical studies (16), while oral hydrolyzed ESM has been shown to improve skin and hair appearance in healthy adults and to promote keratinocyte differentiation and skin structural regulation in experimental models (17, 18). In addition, nutrition-related improvements in skin barrier function and antioxidant-associated indices have also been documented in companion animals, supporting the relevance of these endpoints for evaluating integumentary health (14).

Improvement in coat appearance in the present study appears to be underpinned by concomitant remodeling of hair shaft microstructure. In our experiment, supplemented cats exhibited a more uniform cuticle surface and improved scale alignment, and the SH + ESM group showed the lowest cuticle height, a significantly greater hair fiber diameter, and the most pronounced increases in hair gloss, hair compliance, and coat brightness. These changes are biologically meaningful because the cuticle is the outermost layer of the hair shaft and is a major determinant of surface smoothness and texture, while the structural organization of the cortex contributes importantly to fiber strength and mechanical behavior (19). In addition, hair-structure studies have shown that uplifted or roughened cuticles increase light scattering and thereby reduce hair luster, indicating that smoother and more compact cuticular architecture is closely associated with a glossier visual appearance (20). This structural–phenotypic relationship is further supported by evidence that age-related alterations in hair diameter and surface topography are accompanied by changes in tactile perception, suggesting that SEM-detectable improvements in fiber morphology can plausibly translate into better coat feel (21). Notably, this interpretation is also broadly consistent with the randomized placebo-controlled trial of oral hydrolyzed eggshell membrane reported by Kalman et al. (18), in which supplementation improved hair appearance-related outcomes in healthy adults. Therefore, the superior gloss and coat smoothness observed in the SH + ESM group may not simply reflect subjective sensory changes, but may also be supported by measurable improvements in hair structural integrity and surface quality.

Another important finding of the present study was the improvement in skin condition and barrier function, particularly in cats receiving the SH + ESM supplementation. In the current trial, dorsal skin images showed that supplemented cats, especially those in the SH + ESM group, exhibited a smoother skin surface, fewer flakes, and more clearly defined follicular openings, and these qualitative observations were further supported by the lower TEWL values observed on day 28 and day 56. TEWL is widely recognized as a sensitive noninvasive indicator of epidermal barrier integrity, and previous studies in cats have demonstrated that closed-chamber TEWL measurement can reliably reflect changes in skin barrier status under both experimental and clinical conditions (5, 6). Therefore, the sustained reduction in TEWL observed in the SH + ESM group may reflect improved functional integrity of the feline skin barrier, rather than changes limited to coat appearance. This interpretation is also supported by previous nutritional studies showing that oral hyaluronic acid can improve skin hydration, epidermal characteristics, and barrier-related parameters (16, 22). In parallel, eggshell membrane has been reported to promote keratinocyte differentiation and improve skin structural homeostasis, possibly through calcium-related signaling pathways, while clinical studies have also suggested beneficial effects of oral eggshell membrane supplementation on skin elasticity and appearance (17, 23). The more comprehensive effects observed in the SH + ESM group may result from the convergence of several complementary physiological processes. SH is a highly hydrophilic extracellular-matrix component that may improve tissue hydration, viscoelasticity, and water retention. Previous randomized clinical studies have similarly shown that oral HA or SH supplementation can improve skin hydration, epidermal characteristics, and barrier-related parameters, including TEWL (16, 22). In contrast, ESM contains collagen-associated proteins, glycosaminoglycan-related components, amino acids, and other bioactive constituents that may support epidermal structure, extracellular-matrix stability, and hair-fiber formation. Experimental studies have shown that ESM can promote keratinocyte differentiation and increase the expression of structural differentiation markers, while clinical supplementation studies have reported improvements in skin elasticity and hair appearance (17, 18, 23). Thus, SH may primarily contribute to the hydration-related properties of the skin, whereas ESM may provide structural and nutritional support. Their combined administration may simultaneously improve water retention and structural integrity, providing a plausible explanation for the lower TEWL, improved skin elasticity, and better skin surface condition observed in the SH + ESM group.

Beyond the improvements in skin barrier function, the present study also showed that SH + ESM supplementation was associated with a more favorable systemic oxidative and skin-related inflammatory profile. Compared with the control group, cats receiving SH + ESM exhibited lower serum MDA and higher GSH-Px, CAT, and T-AOC, whereas SOD remained unchanged; in parallel, serum IL-31 was significantly reduced in the ESM and SH + ESM groups. Malondialdehyde is a widely used end product of lipid peroxidation, whereas GSH-Px, CAT, and total antioxidant capacity reflect major components of antioxidant defense, and improvement in these markers has been linked with better skin status and barrier recovery in dermatologic conditions (24, 25). The decrease in IL-31 is also noteworthy, because IL-31 is a key pruritogenic cytokine implicated in allergic skin disease; experimental and clinical studies in both dogs and cats have shown that modulation of IL-31 signaling is associated with reduced pruritus and lesion severity (26–28). Therefore, the lower IL-31 concentration observed after ESM-containing supplementation may indicate attenuation of itch-related inflammatory signaling, which could partly explain the concurrent improvements in coat quality and skin appearance. The absence of a significant difference in SOD does not materially weaken this interpretation, as oxidative stress markers do not always change in parallel and CAT, GPx, MDA, and overall antioxidant capacity may be more responsive than SOD in some skin disorders or intervention settings (29). Overall, these findings suggest that the beneficial effects of SH + ESM may be related, at least in part, to reduced lipid peroxidation and a more favorable inflammatory–redox milieu.

The changes in serum and hair composition observed in this study provide an additional mechanistic layer for the improvement in coat and skin quality. Cats receiving SH + ESM showed higher circulating Zn, Cu and VE, together with greater hair Zn deposition and increased levels of several amino acids in hair, including Ala, Val, Met, Leu and His, while Lys, Pro, Arg and Cys were more prominently elevated in the SH + ESM group. These findings suggest that the beneficial effects of SH + ESM were not limited to superficial cosmetic changes, but may also involve improved availability and incorporation of nutrients required for follicular keratinization and hair fiber assembly. In companion animals, skin and coat are highly responsive to nutritional status, and deficiencies of zinc, vitamins, and other essential nutrients can impair keratinization and haircoat quality (30, 31). Zinc is particularly relevant because it is required for epithelial function and keratinization, and recent feeding studies in cats and dogs indicate that trace mineral source can influence haircoat characteristics, hair growth, shedding, and skin-related outcomes (7, 32). Copper may also contribute meaningfully, as copper-dependent enzymes are involved in extracellular matrix stabilization and pigment-related processes in skin and hair (33). In parallel, VE is a major lipid-soluble antioxidant in the skin and has been linked to photoprotection and skin barrier stabilization (34). From the perspective of hair composition, this pattern is also biologically plausible because hair shafts are composed predominantly of keratin, cats and dogs have a particularly high demand for sulfur amino acids, and cysteine is the most abundant amino acid in pet hair; accordingly, increases in Met and Cys, together with Lys, Pro and Arg, may indicate improved substrate support for hair protein synthesis and structural maintenance (15, 35). Therefore, the simultaneous elevation of skin-related micronutrients in serum and structural components in hair supports the view that SH + ESM supplementation enhanced the nutritional substrate base for both epidermal maintenance and hair fiber formation, thereby contributing to the improved coat gloss, compliance, and barrier-related outcomes observed in this study. The combined barrier-supporting effect may also be reinforced by improvements in systemic antioxidant and inflammatory status. The reduction in serum MDA, together with the increases in GSH-Px, CAT, and T-AOC, indicates reduced lipid peroxidation and enhanced antioxidant defense. ESM-derived protein hydrolysates and peptides have previously exhibited free-radical-scavenging, reducing, metal-chelating, and DNA-protective activities *in vitro*, providing a biologically plausible basis for a potential contribution of ESM-associated bioactive components to antioxidant regulation (36, 37). Nevertheless, these findings should be regarded as indirect mechanistic support because ESM-derived peptides and their absorption were not measured in the present study. The more favorable redox environment may help preserve epidermal lipids, cellular membrane integrity, and structural proteins involved in skin and hair formation. In addition, the lower serum IL-31 concentration may reflect attenuation of an inflammatory or pruritic environment that could otherwise adversely affect skin condition. The increased serum concentrations of Zn, Cu, and vitamin E, as well as the increased deposition of hair Zn and several structural amino acids, may further provide nutrients and cofactors required for keratinization, antioxidant protection, protein stabilization, and hair-fiber assembly. Accordingly, the improvements in hair cuticle arrangement, fiber diameter, gloss, and compliance may reflect the integrated effects of enhanced hydration, reduced oxidative damage, moderated inflammatory status, and improved nutrient availability.

Changes in hematological indices in the present study should be interpreted cautiously, because the animals were clinically healthy and the trial was not designed as an inflammatory disease model. The hematological changes observed in this study should be interpreted cautiously in the context of the animals’ physiological condition. All cats were clinically healthy, showed normal growth, and exhibited no treatment-related adverse events, while most leukocyte-, erythrocyte-, and platelet-related indices remained unchanged. The lower granulocyte ratio and granulocyte count observed after supplementation, together with the reduction in serum IL-31, were directionally consistent with a less inflammatory or pruritic systemic environment. Granulocyte-dominant hematological patterns have been associated with inflammatory activation in cats under pathological conditions (38, 39). However, the cats in the present study did not have an inflammatory disease, and individual granulocyte populations and additional systemic inflammatory markers were not evaluated. Moreover, although the absolute granulocyte count in the SH + ESM group remained within the reference interval listed in Table 4, the mean granulocyte ratio was below the corresponding interval. Therefore, the reduced granulocyte indices should not be interpreted as definitive evidence of a systemic anti-inflammatory effect. The higher total red blood cell count, hemoglobin concentration, and mean corpuscular hemoglobin observed in the SH + ESM group were significant relative to the ESM group, but not relative to the control group. These differences therefore do not demonstrate a direct stimulation of erythropoiesis by the combined supplementation. Although reductions in red blood cell- and hemoglobin-related indices are characteristic of feline anemia and may occur during chronic inflammation (40–42), the cats in this study showed no evidence of anemia. In addition, hematocrit did not increase correspondingly, and reticulocyte counts, iron-status markers, erythropoietin, and hydration status were not assessed. The erythrocyte-related findings are therefore more appropriately regarded as evidence of preserved hematological status or normal biological variation rather than a direct hematopoietic effect. Because hematological measurements were available only at the end of the trial and no individual baseline values were obtained, longitudinal studies with larger sample sizes and more comprehensive hematological and inflammatory assessments are required to clarify the physiological significance of these changes.

Dietary supplementation with SH and ESM appeared to influence the feline gut microbiota primarily at the level of community composition rather than overall alpha diversity, with more evident compositional shifts observed in the SH + ESM group. In the present study, neither Shannon nor Chao indices differed significantly among groups, whereas PCoA, LEfSe, and genus-level analyses showed clear treatment-related shifts, with the SH + ESM group exhibiting a more concentrated community pattern and the most prominent enrichment of Blautia- and Lachnospiraceae-associated taxa; Sellimonas was also relatively more abundant in the ESM and SH + ESM groups. Such a pattern is biologically meaningful because, in cats and dogs, diet is a major determinant of gastrointestinal microbial composition and function (43), alpha diversity metrics alone do not necessarily reflect microbiome functional status or host relevance (44). The enrichment of Blautia is of particular interest, as Blautia has repeatedly been linked to a more favorable gut environment, and experimental work has shown that oral administration of *Blautia wexlerae* can exert anti-inflammatory effects while remodeling the intestinal microbial ecosystem (45). Likewise, Sellimonas intestinalis has been proposed as a potential biomarker of intestinal homeostasis recovery (46). Therefore, the increased abundance of Blautia-, Sellimonas-, and Lachnospiraceae-related taxa after SH + ESM supplementation may reflect a shift toward a more functionally favorable microbial configuration. This interpretation is also relevant to the skin-related outcomes observed here, because increasing evidence supports the existence of a gut–skin axis, through which gut microbial alterations may influence epithelial homeostasis and inflammatory skin phenotypes (47). Overall, these data suggest that the microbiota response to SH + ESM was not characterized by a simple expansion of diversity, but rather by selective enrichment of taxa more commonly associated with microbial homeostasis and host benefit.

Spearman correlation analysis in the present study further supports a potential gut–skin axis mechanism underlying the beneficial effects of SH + ESM supplementation. Specifically, *Blautia*, *Sellimonas*, and *unclassified_f__Lachnospiraceae* were positively correlated with serum VE, Zn, and Cu, and in some cases with T-AOC, while showing negative associations with MDA; by contrast, several other taxa, including Ligilactobacillus, were linked to less favorable antioxidant- or micronutrient-related profiles. These findings should not be interpreted as evidence of causality, but they are directionally consistent with the current concept of the gut–skin axis, in which gut microbial communities influence skin physiology through microbial metabolites, immune signaling, and barrier-related pathways (48, 49). This interpretation is also plausible at the taxonomic level. Blautia has recently been shown to maintain colonic mucus function through the production of acetate and propionate (50), whereas Sellimonas intestinalis has been proposed as a biomarker of gut homeostasis recovery (46). In addition, Lachnospiraceae-related taxa are widely regarded as important SCFA-associated commensals with relevance to gut barrier integrity and host immune regulation (51, 52). Because SCFAs can strengthen epithelial tight junctions, enhance mucus function, and modulate inflammatory and oxidative processes (51, 53), the positive association of these taxa with antioxidant- and micronutrient-related indices suggests that the improved skin and coat phenotype observed after SH + ESM supplementation may be partly mediated by microbiota-driven regulation of the host redox and barrier milieu. Nevertheless, this remains a correlative inference, and future studies integrating fecal metabolomics, SCFA quantification, and skin molecular markers will be required to verify this proposed gut–skin axis mechanism. The enrichment of Blautia and Lachnospiraceae-associated taxa in the SH + ESM group may represent an additional microbiota-associated component of this response. Previous experimental evidence has shown that *Blautia coccoides* can produce acetate and propionate and support intestinal mucus function, indicating that members of this genus may exert biologically relevant effects through microbial metabolites (50). The correlations observed in the present study between these taxa and antioxidant- or micronutrient-related indices therefore suggest a possible relationship between intestinal microbial composition and the systemic environment supporting skin and coat health. However, fecal SCFAs and other microbial metabolites were not measured in the present study, and the observed taxonomic shifts therefore cannot establish a metabolite-mediated gut–skin mechanism. Future studies should integrate standardized fecal SCFA quantification with skin barrier, inflammatory, and metabolomic assessments to determine the functional significance of these microbial changes. Moreover, because the study did not include dose-matched 125 mg/kg SH and ESM monotherapy groups or a factorial interaction analysis, the present findings support complementary or potentially interactive effects rather than formally demonstrating statistical or molecular synergy. Further studies using dose–response and factorial designs, together with measurements of epidermal barrier-related proteins, oxidative and inflammatory signaling, microbial metabolites, and skin metabolomic profiles, are required to verify the proposed mechanism.

Several limitations of the present study should be acknowledged. First, the study included a relatively small cohort of healthy 6-month-old Ragdoll cats. Although the use of a homogeneous population reduced biological variability, it also limits the extrapolation of the findings to other breeds, different life stages, and cats with dermatological or systemic disorders. Therefore, the present results should be interpreted as evidence of nutritional effects in healthy juvenile Ragdoll cats rather than as evidence of therapeutic efficacy in cats with clinical skin disease. Second, only one supplementation level was evaluated for SH and ESM, and the combined treatment was administered at a fixed 1:1 ratio. Consequently, dose–response relationships, the minimum effective dose, the optimal SH ratio, and possible additive or synergistic interactions between the two ingredients could not be determined. Third, the 56-day intervention provides information primarily on short-term responses and cannot establish the durability of the observed effects, long-term safety, or changes following supplementation withdrawal. Future studies should therefore include larger and more diverse feline populations spanning multiple breeds and age groups, as well as cats with well-defined dermatological conditions. Multiple supplementation levels and SH ratios should be evaluated using appropriately powered dose–response or factorial designs, together with longer intervention and post-treatment follow-up periods. The integration of longitudinal TEWL measurements, skin barrier-related molecular markers, fecal short-chain fatty acid quantification, and metabolomic analyses would also help clarify the underlying mechanisms and clinical relevance of SH and ESM supplementation.

## 5 Conclusion

In conclusion, dietary supplementation with SH and ESM improved skin and coat health in healthy Ragdoll cats, with more pronounced benefits observed when both ingredients were provided together. Supplementation was associated with improved coat appearance and hair microstructure, reduced TEWL, enhanced antioxidant status, decreased serum IL-31 levels, increased skin- and hair-related nutrient levels, and altered fecal microbial composition. These findings suggest that SH and ESM may support feline skin and coat health by modulating barrier function, oxidative balance, nutritional status, and the gut microbiota. Further studies are needed to confirm these effects in cats with dermatological disorders and to elucidate the underlying mechanisms.

## Data Availability

Sequencing datasets for 24 fecal samples in our study have been deposited into NCBI’s Sequence Read Archive (SRA) database with the BioProject number: PRJNA1462552.
